# Editorial

**Published:** 1861-08

**Authors:** 


### Editorial.

### "FILLING MOLAR TEETH—APPROXIMAL SURFACES."

IN the Southern Dental Examiner for June there is an article on filling cavities in the proximal surfaces of the molar teeth, which, in two or three respects, it may not be amiss to notice. In the first place, the method of filling there described is not new, but is practiced by many, and has been described, but perhaps not just in the language here used. It is not objectionable, however, to describe an operation, though it may have been described before; especially if any new phase or point can be presented, or even the old ones be made to assume a new and attractive dress. The objectionable feature is a presentation of this kind, and denominating it "*my mode of filling.*" This style of speaking always conveys the idea that the method is "*mine*" by right of discovery.

There are two extremes in this matter: a man gets an idea, he studies it, and evolves it till he imagines it originated with himself—the other extreme is exhibited, when something really new and valuable is discovered and described by some diligent investigator, then some one, more wise than "any other man," announces that he has known all about that long ago.

The method of preparing cavities, as described by Dr. S., is good, at least in those cases where the lateral walls of the cavity are firm and not liable to be broken away in the operation of filling, or by subsequent accident. But this method of introducing the filling is not the best; it can hardly be said to be good; for he says "the filling is liable to come out unless the operation is done with a master hand." Can such a method be called a good one? It is certain if the "*surface face*"—face-face—(where is the face that is not a surface face?) becomes broken, the filling will very soon be destroyed. Into such a cavity, if a filling of adhesive gold be introduced with care and an *ordinary* amount of skill, though it be not "with a master hand," it will not come out till the walls of the cavity are broken away from it.

There is another feature of this article which it may be well to notice, and that is the very free use of the personal pronouns *I* and *my*. Dr. S. is not the only member of the dental profession who indulges in this direction, but there is a bountiful supply of *No.* 1 personal pronouns in this article; on the first page there are twenty-three, and in nine lines of this page I and my occur ten times; this style of writing is usually regarded as indicative of egotism. This little word may with propriety be used occasionally; but to thrust it in at every point where it can be introduced, is pretty good evidence that the writer stands well in his own estimation.

The following is in rather bad taste, to say the least: I prepare my gold foil, I take No. 6. The idea would have been quite as clear, and less objectionable, thus. Now prepare the gold foil, No. 6.

In the article under review, the objectionable frequency of this little word could have been obviated by a little attention on the part of the writer, if he had been disposed to do so. Dr. S. is not a sinner above all men in this respect; there are others worse than he. Some of the discussions of the dental associations are shamefully egotistical. *I* take *my* file and separate *my* tooth ; then take *my* excavator and clean out *my* cavity. *I* make in *my* cavity *my* retaining points at the bottom. *I* prepare *my* gold for *my* filling in the form of blocks ; *I* take up *my* gold on the point of *my* instrument and place it in *my* cavity.

Now, any one would say this looks silly in print ; well it does, but then it hardly comes up to the point of egotism sometimes exhibited in discussions in dental meetings. It is true that the *agony* is not brought out in full in the printed reports.

After an article is written, just strike out all the first personal pronouns, and see if it does not look quite as well. Three or four on a page is admissible, but when it comes to thirty or forty, it is abominable. T.

### FISTULOUS OPENING THROUGH THE CHEEK.

Mr. N , aged twenty five, called to have a tooth extracted, on account of periostitis and incipient abscess. The tooth, a second lower molar, was easily removed, and but little hemorrhage followed its extraction. The tumefaction, however, continued and increased. A lead cap was fitted over the tumor, and pressure kept up, by means of lint and collodian strips. The swelling disappeared, and the cap was removed.

A month after the tooth was extracted, a small white tumor appeared on the outside of the cheek, and in a few days discharged a thick, unhealthy pus. About six weeks later, my attention was again called to the case, when I was surprised to find that there was nothing like granulations in the socket. Water, injected into it, passed entirely through the cheek. Indeed, a fine probe was readily passed into the opening in the cheek, and up through the socket.

Having satisfactory evidence that there was no venereal taint, I concluded to rely, at least for a time, on local treatment.

The fistula was washed out with tepid water once a day ; and, about twice a week, tincture of iodine was freely applied to its entire surface. The treatment was continued for two weeks, when the appearances were so favorable that the case was left to nature, watching closely, however, for any unfavorable symptoms. In four weeks from the commencement of the treatment, all was well.

This patient had always a weak constitution—was a printer, and was well marked with that pallor peculiar to his craft. The powers of life being feeble, active and frequent local medication would have probably destroyed rather than built up the parts to which it was applied. This remark is made from the known fact that many are in the habit of making daily applications of even active escharotics, in such cases. W.

### EXCURSION TO CHICAGO.

On the 24th ult., about twelve hundred of our citizens started on an excursion to Chicago, over the Cincinnati and Chicago Air Line Railroad, by invitation. The excursionists occupied three trains, consisting of twenty-two cars. They passed smoothly, safely and pleasantly over the road from Cincinnati to Chicago in about twelve hours, one hour more—owing to the heavy trains—than the running time. A portion of the road has recently been built, and is not as smooth as a well kept old road; but that will soon be remedied, for it is being ballasted, and will be in perfect condition, as soon as the new portions of the road become thoroughly settled.

So far as the travel between Cincinnati and Chicago is concerned, this road has all the advantages of any other, and some that no other route has or can attain. There are no heavy grades, and the road is the straightest over which we have ever traveled; almost the whole distance, the road is without any perceptible curve. These two conditions lessen very greatly the liability to accident; both curves and strong grades are hard on machinery, wear it rapidly, and render breakage far more liable; the road, too, is far more likely to get out of repair.

Again, the running time on this road is two hours less than by any other; this is not so by greater speed, but the road is forty-two miles shorter than any other, so that the same speed being made on this, as on the other routes, more than two hours would be gained.

Close and certain connections are made with other roads extending or radiating from it. Care for the comfort and safety of the passengers is a prominent idea with the officers. This road must, if rightly managed, so soon as it becomes known, take all the direct travel between the two cities.

We refer to this road, because we think those who travel should know the safest and most direct routes between important points.

T.

### CLOSURE OF THE PAROTID DUCT.

Last week, Miss H. complained of fullness and slight soreness of the left cheek. On examination, the teeth were found healthy and comfortable. A more minute examination revealed the fact that the parotid duct was closed. At the side of its late outlet was a tumor the size of a pea. I transfixed this with a curved bistoury, and divided it into two flaps, and proceeded to explore with a probe, expecting to find the duct obstructed by a small calculus. Being unable to penetrate the duct to any extent, the flaps of the tumor were divided, making a conical incision ; and the patient was directed to use fomentations, and to keep the general health in as good condition as possible, hoping that, with the abatement of the local inflammation, the duct would become pervious. If it does not, what shall I do ? Do tell, kind reader. W.

### DISSOLUTION OF THE UNION.

No reference to politics, good reader, nor to the state of the country ; for this is the Dental, not the *Political* Register. But our profession has been long familiar with, and benefited by another union—the union of the talents and business energies of Messrs. Jones, White & McCurdy. And this union is dissolved. As our readers know, it was partly dissolved, some time ago, by the withdrawal of Mr. M'Curdy ; but the title, thus changed, did not sound so strangely, for, as long names are commonly abbreviated, we were sometimes content to *say* " Jones & White," and *think* " McCurdy." But now—that " threefold cord, not easily broken," is untwisted, and each strand is a string by itself. Now we are done with Jones & White's teeth, Jones & White's foil, Jones & White's operating chairs, Jones & White's everything a dentist wants. Now, when we visit Philadelphia, New York, or Boston, the friends we leave behind us will no longer address us, in parenthesis, "Care of Jones & White;" for "Jones & White" *non est.*

Jones, White & McCurdy—Jones & White. Why, our pen likes to write it! We made calculations for *some* changes—expected that comets would come, that empires would decay, and new ones spring from their dust. Little things like these we looked for; but we didn't expect to be a member of our profession without any "Jones & White." But the thing has come to pass. Things will happen sometimes: and they appear to happen much faster, and a great deal oftener, now that the old man of the hour-glass and scythe has silvered our foretop with his icy touch, than they did when our locks were flaxen, and our brow unwrinkled. Then it would have taken the man of destiny a long time to do away with "Jones & White;" but now he drives in a gallop, and that is why we are out of a "Jones & White."

Well—if we haven't the article, we still have the stuff it was made of; and so, Long life to Jones, to White, and to McCurdy.

W.

### MICROSCOPIC.

In the preservation of teeth or other animal tissue for microscopic examinations, the chief object is to keep them in a natural condition. To effect this, they should not be allowed to dry or decompose in any way or degree; this may be secured by at once bottling them in whisky or other spirit of equal strength, or a saturated solution of common salt. Soft tissue in solution of half this strength will be preserved with less change. T.

American Agriculturist, for the Farm, Garden and Household.

This is a monthly journal of agriculture, of 32 pages, published by Orange Judd, New York. It contains more matter than any other agricultural paper in our country. All subjects pertaining to agriculture and horticulture are discussed in the most thorough and practical manner. The most common-place subjects are taken up and treated so as to elicit new interest and attention. The topics of discussion are presented in such a manner as to be attractive and interesting to all classes of persons.

Almost any one who takes it up will examine it throughout. This is just the paper by which to become posted in all those little matters pertaining to agriculture and horticulture that are interesting to all classes of persons. This paper should be in every family; for from it a fund of knowledge may be gained that will be useful in a great many ways, and to every body. It is published at one dollar per annum. T.

